# Neuroprotection of IGF-1 in neonatal hypoxic-ischemic brain injury through downregulation of FoXO3a-PUMA pathway

**DOI:** 10.3389/fncel.2025.1685800

**Published:** 2025-11-03

**Authors:** Yanli Tang, Rui Zhong, Jiayi Liang, Shuang Liu, Wanxia Liu, Tao Liu, Baohong Yuan, Mengya Jiao, Hui Yin

**Affiliations:** 1 Affiliated Shenzhen Women and Children’s Hospital (Longgang) of Shantou University Medical College (Longgang District Maternity & Child Healthcare Hospital of Shenzhen City), Shenzhen, China; 2 Guangdong Provincial Key Laboratory of Pharmaceutical Bioactive Substances, Guangdong Pharmaceutical University, Guangzhou, China; 3 Department of Microbiology and Immunology, Guangdong Pharmaceutical University, Guangzhou, China; 4 Department of Rheumatology and Clinical Immunology, Jiangxi Provincial People’s Hospital, The First Affiliated Hospital of Nanchang Medical College, Nanchang, China; 5 Jiangxi Province Key Laboratory of Immunology and Inflammation, Jiangxi Provincial People’s Hospital, Nanchang, China; 6 JXHC Key Laboratory of Rheumatology and Immunology, Jiangxi Provincial People’s Hospital, Nanchang, China

**Keywords:** IGF-1, neonatal hypoxic-ischemic encephalopathy, neuroprotection, FoXO3a, PUMA

## Abstract

Insulin-like growth factor-1 (IGF-1) is a single chain polypeptide hormone that plays an essential role in intrauterine and postnatal growth. Recent studies suggest that IGF-1 and its receptor IGF-1R are involved in the pathogenesis of neurological diseases. Here, we explore the effect of IGF-1 signaling in neonatal hypoxic-ischemic (HI) brain injury and elucidate the underlying mechanisms of action. We found that the expression levels of IGF-1 were markedly enhanced in astrocytes post HI. Delivery of IGF-1 significantly alleviates neonatal brain insult and improves neurobehavioral disorders in neonatal mice after HI challenge. Through binding to IGF-1 receptor (IGF-1R), IGF-1 inhibited the apoptosis of neuronal cells following HI exposure. IGF-1 improved neuronal cell survival and proliferation through activation of phosphorylated AKT signaling. Of note, the protective property of IGF-1 against ischemic neuronal insults was dependent on suppression of the FoXO3a-PUMA signaling pathway. Taken together, these findings suggest that IGF-1 may represent a new neuroprotectant for newborns with hypoxic-ischemic encephalopathy.

## Graphical Abstract

**Figure:**
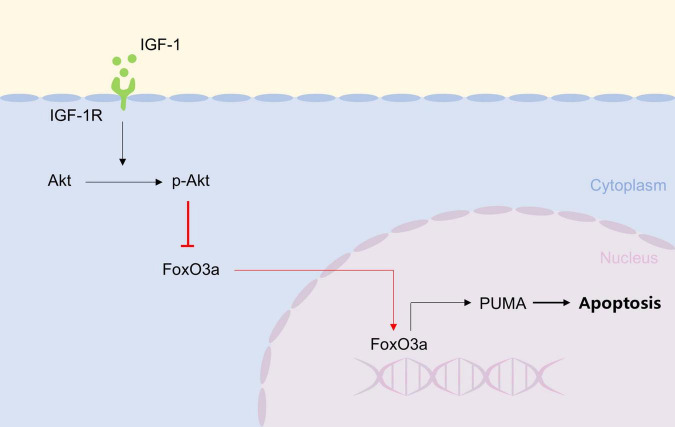
IGF-1, by binding to IGF-1R, activates AKT to inhibit FoXO3a nuclear translocation, thereby preventing its binding to the PUMA promoter and subsequent neuronal apoptosis.

## 1 Introduction

Neonatal hypoxic-ischemic encephalopathy (HIE) is one of the major forms of neonatal brain injury and the most common cause of death and disability in human newborns (Mohsenpour et al., 2021). In addition to the intuitively apparent loss of brain volume, there are multiple molecular mechanisms leading to cell death (Li et al., 2022; Tao et al., 2024). Although there have been major advances in modern technology and an increased understanding of fetal and neonatal pathologies, the therapeutic interventions for HIE in the clinical setting are limited (Babbo et al., 2024; Yang et al., 2023). Thus, it is urgently needed to develop safe and effective therapies for the treatment of neonates suffering HIE.

Insulin-like growth factor-1 (IGF-1) is a single chain polypeptide hormone that plays an essential role in intrauterine and postnatal growth (Sádaba et al., 2016; Yan et al., 2022). IGF-1 and its receptor are widely expressed in brain tissue cells, and it is possible that it is involved in early central nervous system (CNS) development and neuronal plasticity (Bhalla et al., 2022; Ballester-Rosado et al., 2025). IGF-1 is essential for brain development and has been strongly implicated in post-injury repair and neuronal survival. IGF-1 has a constructive significance for normal brain growth and development, and may play a role in protection and repair after brain injury (Montivero et al., 2021). Astrocytes, as key homeostatic cells in the CNS, play a critical role in neuroinflammation and trophic support after brain injury, making them a prime candidate for investigating endogenous neuroprotection. The role of serum IGF-1 level in clinical judgment of the severity of neonatal HIE and prognosis has been reported (Janowska et al., 2020; Umran et al., 2016; Kartal et al., 2016). However, the cellular source and precise molecular mechanism of IGF-1 in the injured neonatal brain remain unclear. This study was therefore designed to define the role of astrocyte-derived IGF-1 and its downstream pathway in neonatal HI injury.

In the present study, we observed that administration of IGF-1 remarkably attenuated HI-mediated neonatal brain damage, whereas blockage of IGF-1 receptor (IGF-1R) signaling exacerbated brain infarction and behavioral disorders. Significantly, the neuroprotective effect of IGF-1 on ischemic neuron survival was involved on downregulation of the FoXO3a-PUMA signaling pathway. We hypothesized that IGF-1, derived primarily from astrocytes, protects against neonatal HI brain injury by activating neuronal survival signaling and suppressing the FoxO3a-PUMA apoptotic pathway.

## 2 Materials and methods

### 2.1 Mice and treatment

C57BL/6J mice were obtained from the Center of Experimental Animals of Guangdong Province and housed in specific-pathogen-free conditions. One male and two females were mated for reproduction, and their pups at 7 days old (P7) were chosen for HI operation. All experiments were conducted in accordance with ARRIVE guidelines (Kilkenny et al., 2010) and approved by the Animal Care and Use Committee of Guangdong Pharmaceutical University. Recombinant mouse IGF-1 was purchased from Sino Biological Inc., Mice were injected i.p. with IGF-1 (0.2 μg per mouse) or PBS for 3 days after HI operation. For Picropodophyllin (PPP, an IGF-1 receptor inhibitor; MedChemExpress) treatment, mice were injected i.p. with PPP (one hour before IGF-1 or PBS administration) for 3 days after HI operation. Sample sizes for animal experiments were determined based on our previous studies using the same HI model and preliminary experiments, with group sizes of no less than six animals to ensure robust statistical power. Animals were randomly assigned to experimental groups, and investigators were blinded to group allocation during data collection and analysis.

### 2.2 Neonatal hypoxia-ischemia model

The modified Rice-Vannucci model was used to establish HI brain injury (Rice et al., 1981). P7 mouse pups were anesthetized and underwent a unilateral left common carotid artery occlusion using bipolar electrical coagulation (Vetroson). The incision was cemented with a tissue adhesive (3M Vetbond). After a recovery period of 2 h, pups were placed in a hypoxia chamber (containing 8% oxygen in a balance with 92% nitrogen) maintained at 37 °C for 90 min. Pups were then recovered for 30 min and returned to their dams. The sham control mouse pups were mock-treated with a small incision in their neck without artery occlusion and placed in a chamber at normal air temperature.

### 2.3 Neurobehavioral evaluation

Mouse pups in each treatment group were subjected to three neurobehavioral tests at 7 days after HI or sham operation: (1) geotaxis reflex for diagnosing the function of vestibular and proprioception; (2) the forelimb grip test for evaluating grip force and fatigability; and (3) cliff avoidance reaction for assessing the ability of rodents to respond to adverse environments (Xiao et al., 2014). All the experiments were observed by at least two naive lab workers, and all animal groups maintained the same testing methods.

### 2.4 Nissl staining

Mouse pups were anesthetized and transcardially perfused with 4% PFA in PBS. The brain tissues were fixed with 4% PFA for 24 h, embedded in paraffin, and sliced coronally to 5 μm thick. The sections were deparaffinized in xylene and rehydrated in 100%–70% gradient ethanol. Then, they were stained with Nissl staining solution. The slices were washed with double-distilled water, dehydrated in ethanol, cleaned with xylene, and examined with an Olympus BX51 microscope. Nissl bodies are large and numerous, which indicates that neurons have strong functions of synthesizing proteins. When neurons are injured, the number of Nissl bodies decreases or even disappears. An expert in the field of pathology, blinded to allocation, assessed the sections for Nissl staining.

### 2.5 Primary mouse cortical neuron cultures

Primary mouse cortical neurons were cultured as described previously (Jiao et al., 2020, 2021). Briefly, cortices from newborn mouse pups were dissected and pooled. After mechanical and chemical dissociation, dissociated cells were planted onto a poly-L-lysine-coated culture plate in neurobasal media (Gibco, United States) containing 2% B27 (Gibco, United States). Cells were cultured in a humidified incubator (95% O_2_ and 5% CO_2_) at 37 °C for 7 days before experiments. Cell culture experiments were performed in three independent biological replicates, a sample size determined to be sufficient based on consistent and significant effects observed in our previous work (Jiao et al., 2020, 2021) and preliminary data for the current assays.

### 2.6 Oxygen-glucose deprivation/reoxygenation (OGD/R) challenge

The media of neuron cultures were replaced with the glucose-free DMEM and transferred into an anaerobic chamber (5% CO_2_ and 95% N_2_) at 37 °C for 3 h. The neurons were incubated again in neurobasal medium containing 2% B27 and returned to the normoxic conditions for another 24 h. For IGF-1 treatment, cultured cortical neurons (5 × 10^5^) were treated with IGF-1 (25 ng/mL) throughout the whole period of OGD/R, with or without pretreatment of the PI3K inhibitor Ly294002 (Ly29, 1 μM; Selleck). Cells without exposure to OGD/R were defined as the control group. The cells were collected after OGD for the following experiments.

### 2.7 Lentivirus transduction

Primary cortical neurons were infected with either FoxO3a-shRNA lentiviral particles (FoxO3a-shRNA LV) or control shRNA LV (Santa Cruz biotechnology) in neurobasal media with 5 μg/mL polybrene at multiplicities of infection (MOI) of 15. After 12 h of culturing, the medium was replaced by fresh neurobasal media containing 2% B27 in order to remove debris and inactive lentiviruses.

### 2.8 Cell viability assay

Cell viability was assessed by cell counting kit-8 (CCK-8; Dojindo, Japan) according to the manufacturer’s instructions. Cells were seeded at a density of 5 × 10^4^ cells per well in 96-well plates. After undergoing OGD/R, the cells were cultured with IGF-1 (25 ng/mL), or both IGF-1 and Ly294002 (1 μM) for 24 h and subsequently with CCK-8 solution for 2 h at 37 °C. The optical absorbance at 450 nm was detected using a microplate reader (Model 680, Bio-Rad Laboratory).

### 2.9 Flow cytometry

To examine the percentages of GFAP^+^ or NeuN^+^ cells, cells were stained with a primary anti-GFAP (Sigma) or anti-NeuN antibody (Sigma) followed by PE- or FITC-conjugated secondary antibodies (eBioscience). To examine the percentages of IGF-1^+^ or IGF-1R^+^ cells, cells were stained with a primary anti-IGF-1 (SinoBiological) or anti-IGF-1R antibody (R&D Systems) followed by PE- or FITC-conjugated secondary antibodies (eBioscience). For analysis of cell proliferation, cells were fixed and permeabilized using the BD Cytofix/Cytoperm kit, and stained intracellularly with PE anti-Ki-67 (eBioscience). Cell apoptosis was measured using in situ apoptosis detection kit (Roche) according to the manufacturer’s directions. For analysis of phosphorylation Akt-S473, cells were fixed and permeabilized with the BD Cytofix/Cytoperm kit (BD Biosciences) and then stained with anti-phospho-Akt-S473 PE or isotype control (eBioscience). For analysis of PUMA protein levels, cells were fixed and permeabilized in 0.25% saponin. Cells were stained with a primary anti-PUMA antibody (Abcam) followed by PE-conjugated secondary antibody (eBioscience). Flow cytometric analysis was performed with a FACSCalibur cytometer (BD Biosciences) and CellQuest v3.3 software.

### 2.10 Immunofluorescence

Brain sections were dewaxed, and incubated with 10% BSA for 1 h, and then stained overnight at 4 °C with either anti-GFAP (Sigma) or anti-NeuN (Sigma) and with either anti-IGF-1 antibody (SinoBiological) or anti-IGF-1R antibody (R&D Systems). On the following day, sections were incubated with Alexa Fluor 488- or Alexa Fluor 555-conjugated secondary antibodies (Abcam) for 1 h. TUNEL staining was performed with in situ cell death detection kit (Roche). The slides were counterstained with nuclear dye DAPI and observed on an Olympus BX51 microscope.

### 2.11 Western blotting

Total proteins from ischemic brain tissue and primary neurons were extracted by tissue homogenization in RIPA buffer containing a proteinase inhibitor cocktail (Santa Cruz Biotechnology). The cytoplasmic and nuclear extracts of neurons were prepared using a cytoplasmic and nuclear protein extraction kit (Beyotime Biotech Inc.) following the manufacturer instructions. Equal amounts of lysed and boiled protein (10 μg/well) were separated on 12% SDS-PAGE gel and proteins were transferred onto polyvinylidene difluoride (PVDF) membranes. The membranes were incubated with primary anti-Bax (Boster Biological Technology), anti-Bcl-2 (Thermo Fisher Scientific), anti-PUMA (Abcam), anti-Akt (Cell Signaling Technology), anti-phospho-Akt (Cell Signaling Technology), anti-FoxO3a (Cell Signaling Technology), anti-β-actin (Abcam) or anti-Histone H3 (Abcam) antibodies, and then with HRP-conjugated secondary antibody. Images were analyzed using Image J and normalized to β-actin or Histone H3.

### 2.12 Real-time PCR (RT-PCR) analysis

Total RNA from cerebral hemisphere on the ischemic side was extracted with TRIzol reagent (Invitrogen) and reverse transcription was performed using the first strand cDNA synthesis kit (Invitrogen). All RT-PCR reactions were performed with an ABI PRISM^®^ 7000 Sequence Detector Systems (Applied Biosystems). Primer sequences were synthesized by Sangon Biotech (Shanghai, China) and listed as follows: IGF-1 forward 5′-CTG GAC CAG AGA CCC TTT GC-3′, reverse 5′-GGA CGG GGA CTT CTG AGT CTT-3′; IGF-1R forward 5′-CCT GAG GCG TGG AGA TAG AG-3′, reverse 5′-TGT CAG CCC ACC CTA AAA AC-3′; IL-6 forward 5′-TCT GGG AAA TCG TGG AAA TGA G-3′, reverse 5′-TCT CTG AAG GAC TCT GGC TTT GTC-3′; IL-1β forward 5′-GCC CAT CCT CTG TGA CTC-3′, reverse 5′- TGT GCC GTC TTT CAT TAC-3′; TNF-α forward 5′-CAT CTT CTC AAA ATT CGA GTG ACA A-3′, reverse 5′-TGG GAG TAG ACA AGG TAC AAC CC-3′; β-actin forward 5′-CAT CCG TAA AGA CCT CTA TGC CAA C-3′, reverse 5′-ATG GAG CCA CCG ATC CAC A-3′. The results were shown as relative expression values normalized to β-actin.

### 2.13 Electrophoretic mobility shift assay (EMSA)

The nuclear extracts of neurons were prepared using a cytoplasmic and nuclear protein extraction kit (Beyotime Biotech Inc.) following the manufacturer instructions. The sequences of biotin-labeled PUMA probe were as follows: forward 5′-TGG CGG GTT TGT TTA CAA ACA ATG GGG TTC-3′, reverse 5′-GAA CCC CAT TGT TTG TAA ACA AAC CCG CCA-3′. EMSA was performed according to the manufacturer’s protocol (Beyotime Biotech Inc.). Briefly, each EMSA reaction contained 2 μL nuclear extract protein, 1 μL biotin-labeled double-stranded probes, 2 μL 5 × EMSA/Gel-Shift binding buffer and 5 μL nuclease-free water. The reaction mix was incubated for 20 min followed by separation of the protein-DNA complexes using a 4% non-denaturing polyacrylamide gel. Next, the complexes were transferred to a nylon membrane, which are then exposed to ultraviolet light. The protein-DNA bands were detected using streptavidin-HRP conjugate and subsequently visualized with enhanced chemiluminescence reagents.

### 2.14 Statistical analysis

Statistical analyses were performed using SPSS version 18.0 and graphed with GraphPad Prism 7. Statistical differences between groups were evaluated by One-way ANOVA or Student’s *t*-test. Data were presented as the mean ± standard error of the mean (SEM). *P*-value less than 0.05 were considered statistically significant.

## 3 Results

### 3.1 HI injury induces an elevation of IGF-1 in the neonatal brain

**FIGURE 1:**
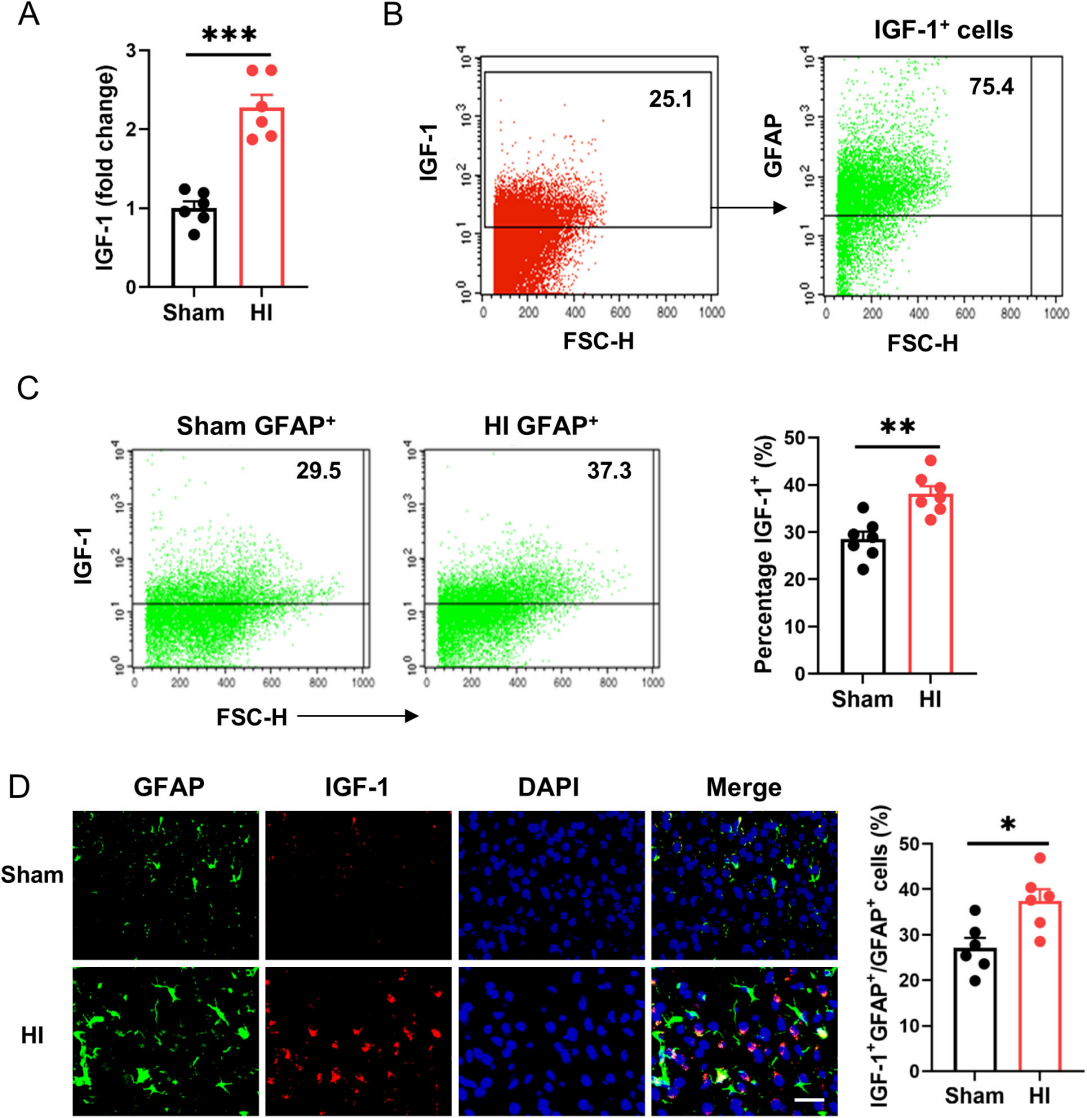
Astrocytic insulin-like growth factor-1 (IGF-1) is robustly upregulated in neonatal brain after hypoxic-ischemic (HI). **(A)** Real-time PCR (RT-PCR) analysis of IGF-1 in neonatal brain at 7 days after HI or sham operation (*n* = 6/group). **(B)** Representative FACS analysis of the proportion of astrocytes in IGF-1 expressing cells from normal neonatal brain. **(C)** Representative FACS analysis (left) and statistical analysis (right) of IGF-1^+^ astrocytes in neonatal brain at 7 days after HI or sham operation (*n* = 6/group). **(D)** Representative images (left) and statistical analysis (right) of GFAP (green) and IGF-1 (red) in the cerebral cortex of mice at 7 days after HI or sham operation (*n* = 6/group). Scale bar, 25 μm. Data are mean ± SEM. Statistical differences between two groups were evaluated by two-tailed Student’s *t*-test. *P < 0.05; **P < 0.01; ***P < 0.001.

To identify the effect of IGF-1 in post-HI neuroprotection, we firstly examined the expression of IGF-1 on the brain after HI injury in neonatal mice. A dramatic increase in IGF-1 mRNA was observed at the lesion site (Figure 1A). Flow cytometry analysis showed that IGF-1 was mainly derived from astrocytes (Figure 1B), and the expression of IGF-1 in astrocytes was further enhanced following HI challenge (Figure 1C). Furthermore, immunofluorescence staining confirmed the expression of IGF-1 in astrocytes, which was elevated after HI exposure (Figure 1D). Together, these results suggest that IGF-1, mainly derived from astrocytes, may be involved in neonatal brain HI injury.

### 3.2 IGF-1 delivery alleviates HI-induced neonatal brain injury

**FIGURE 2:**
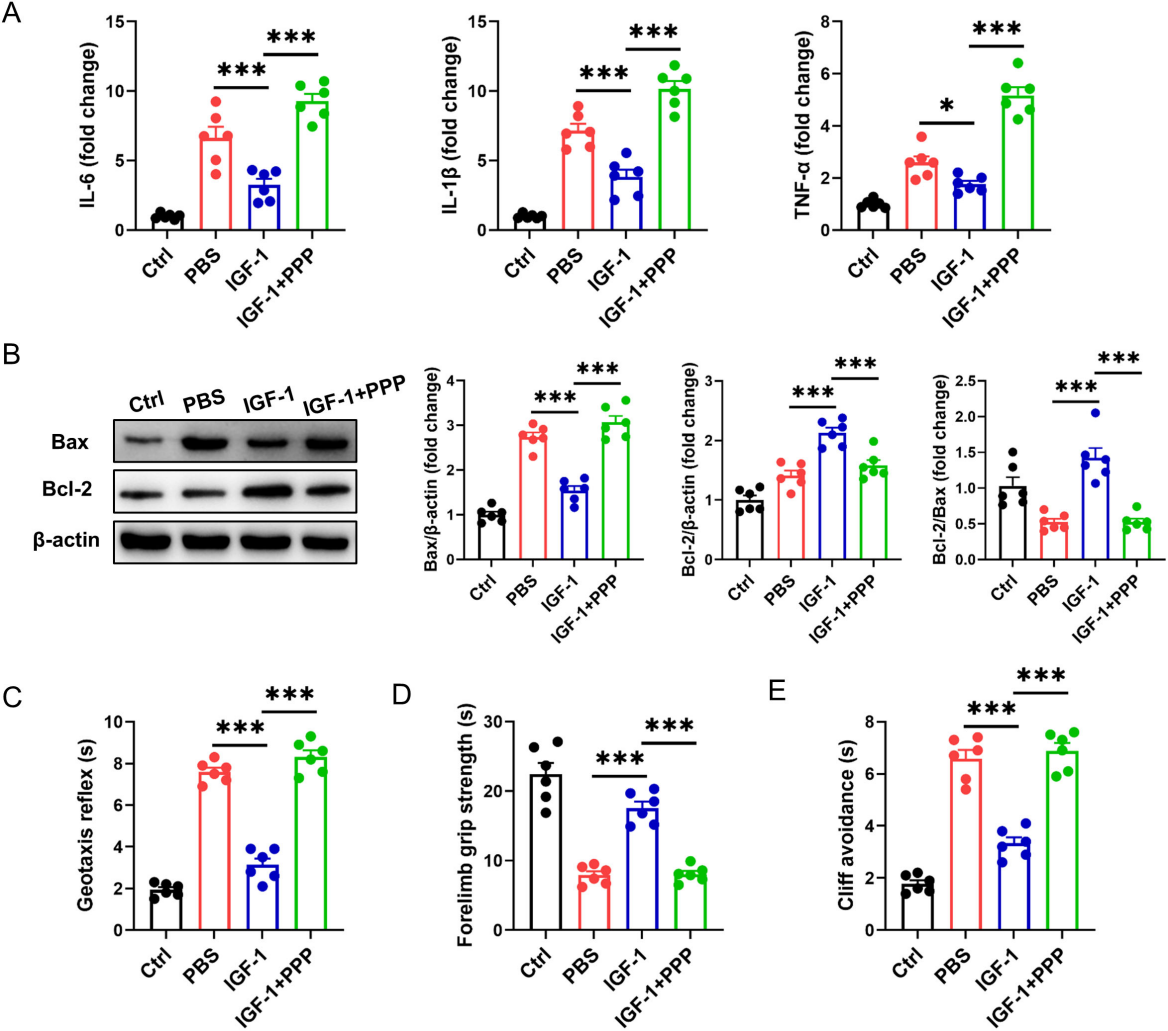
Insulin-like growth factor-1 (IGF-1) ameliorates hypoxic-ischemic (HI)-induced brain injury in neonatal mice. **(A)** Real-time PCR (RT-PCR) analysis of proinflammatory cytokines IL-6, IL-1β, and TNF-α in neonatal brain from IGF-1- and PBS-treated mice at 7 days post HI (*n* = 6/group). **(B)** Representative western blot images (left) and densitometric analysis (right) of Bax and Bcl-2 in neonatal brain from IGF-1- and PBS-treated mice at 7 days post HI (*n* = 6/group). **(C–E)** Neurobehavioral outcomes of the geotaxis reflex **(C)**, forelimb grip test **(D)** and cliff avoidance reaction **(E)** from IGF-1- and PBS-treated mice at 7 days post HI (*n* = 6/group). Data are mean ± SEM. Statistical differences among multiple groups were conducted using One-way ANOVA. *P < 0.05; ***P < 0.001.

Next, we explored whether exogenous IGF-1 can ameliorate brain damage using a neonatal mouse HI brain injury model. Compared with the PBS-treated group post-HI, the IGF-1-treated group exhibited significantly reduced expression of proinflammatory cytokines IL-6, IL-1β, and TNF-α. However, these levels reverted to elevated states following PPP administration (Figure 2A). Meantime, IGF-1 markedly reduced the expression of apoptosis-related protein Bax in brain tissue after HI challenge (Figure 2B). The protein ratio of Bcl-2/Bax was obviously restored in IGF-1-treated group compared to the PBS-treated group, which was inhibited by IGF-1R inhibitor PPP treatment (Figure 2B). Neurobehavioral tests evaluating the geotaxis reflex, forelimb grip and cliff avoidance demonstrated that mice in the PBS group displayed significant neurobehavioral disorders post-HI (Figures 2C–E). Delivery of IGF-1 clearly improved these neurological defects, but abrogated after PPP treatment (Figures 2C–E). Thus, these data indicate that IGF-1 protects against brain damage and neurobehavioral deficits in neonatal mice after HI brain injury.

### 3.3 IGF-1R is expressed on neurons under physiological and pathological conditions

**FIGURE 3:**
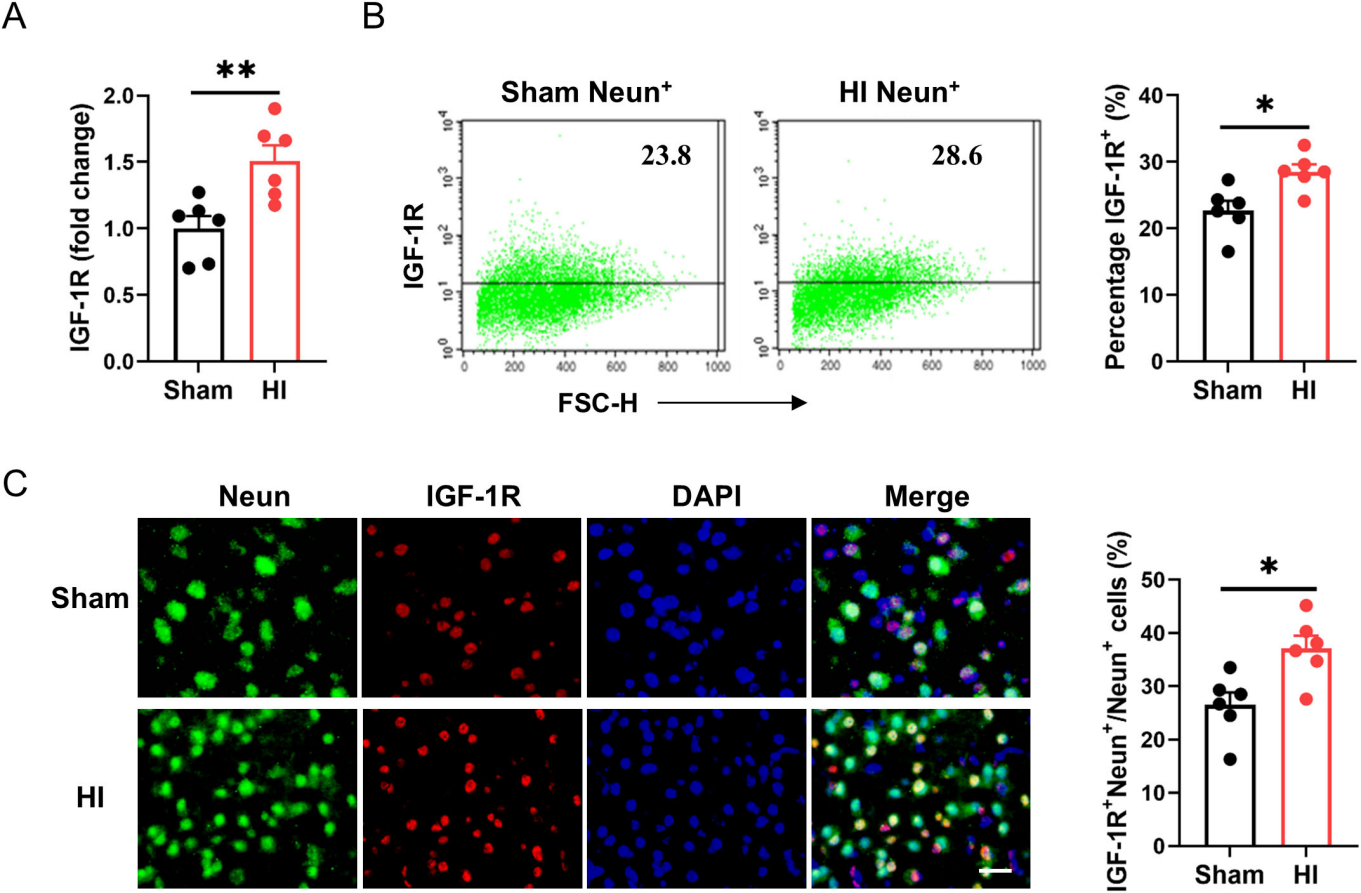
Insulin-like growth factor-1 (IGF-1R) expression on neurons is elevated after hypoxic-ischemic (HI) challenge. **(A)** Real-time PCR (RT-PCR) analysis of IGF-1R in neonatal brain at 7 days after HI or sham operation (*n* = 6/group). **(B)** Representative FACS analysis (left) and statistical analysis (right) of IGF-1R^+^ neurons in neonatal brain at 7 days after HI or sham operation (*n* = 6/group). **(C)** Representative images (left) and statistical analysis (right) of Neun (green) and IGF-1R (red) in the cerebral cortex of mice at 7 days after HI or sham operation (*n* = 6/group). Scale bar, 25 μm. Data are mean ± SEM. Statistical differences between two groups were evaluated by two-tailed Student’s *t*-test. *P < 0.05; **P < 0.01.

To determine the cellular targets of IGF-1 signaling in the brain, we used qPCR and flow cytometry to analyze the expression of the IGF-1R on neurons in the normal and the ischemic brain after HI exposure. As shown in Figure 3A, IGF-1R mRNA levels were elevated at the lesion site. In normal brains, IGF-1R was existed on the surface of NeuN^+^ neurons. Following brain HI challenge, IGF-1R expression levels were significantly increased in this neuronal population (Figure 3B). Immunofluorescence staining confirmed the expression of IGF-1R on Neun^+^ neurons post-HI (Figure 3C). Therefore, the data gathered suggest that IGF-1R is expressed on neurons under physiological conditions and is increased under pathological conditions.

### 3.4 IGF-1 enhances neuron survival after HI injury

**FIGURE 4:**
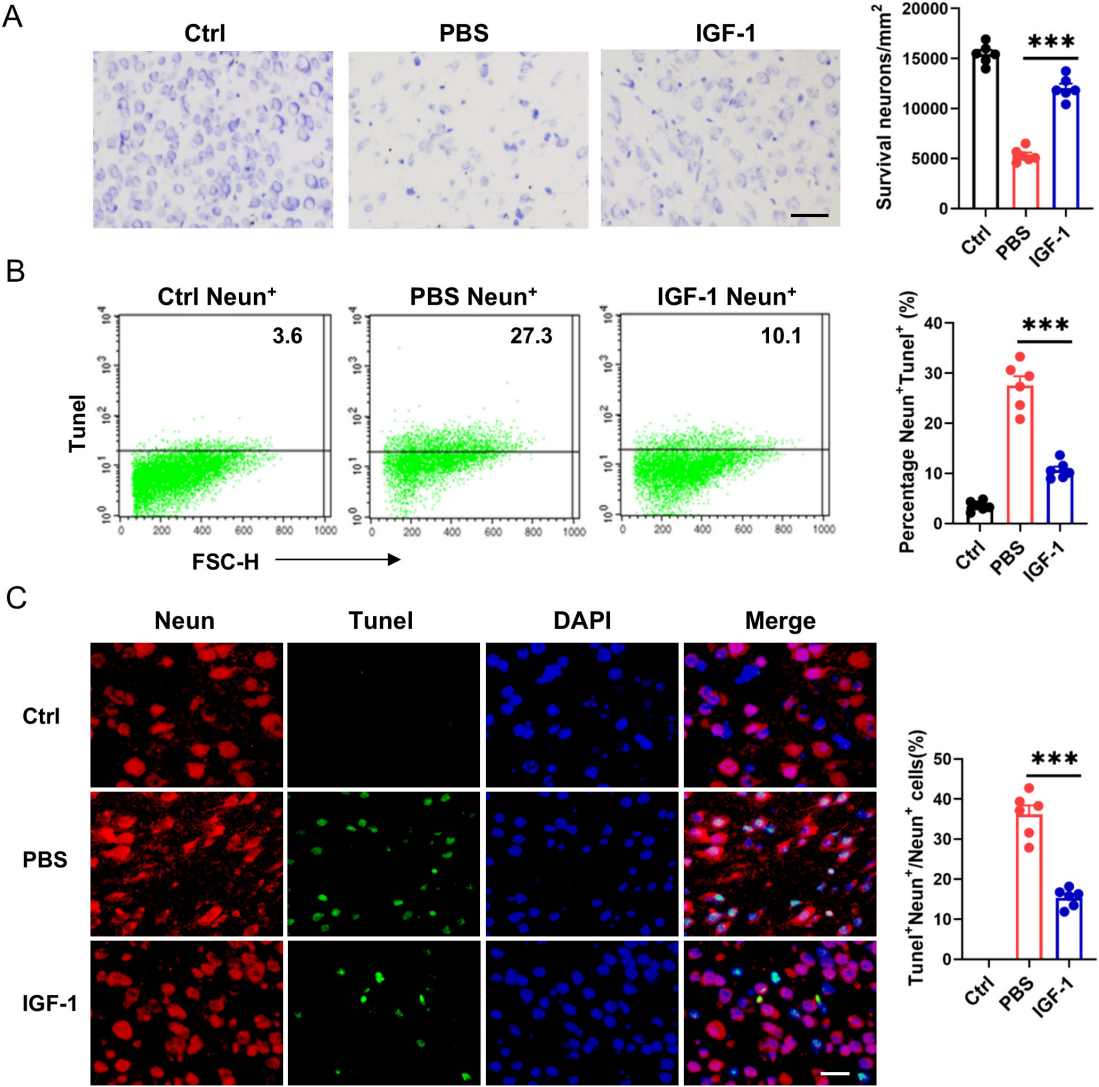
Insulin-like growth factor-1 (IGF-1) promotes neuron survival after hypoxic-ischemic (HI) injury. **(A)** Representative photographs of Nissl staining in the cerebral cortex from every group of mice at 7 days post HI (*n* = 6/group). Scale bar, 50 μm. **(B)** Representative FACS analysis (left) and statistical analysis (right) of Tunel^+^ neurons in neonatal brain from every group of mice at 7 days post HI (*n* = 6/group). **(C)** Representative images (left) and statistical analysis (right) of Neun (red) and Tunel (green) in the cerebral cortex from every group of mice at 7 days post HI (*n* = 6/group). Scale bar, 25 μm. Data are mean ± SEM. Statistical differences among multiple groups were conducted using One-way ANOVA. ***P < 0.001.

As neurons express high levels of IGF-1R under normal conditions and after HI, we assessed the direct effects of IGF-1 on neuron survival. Nissl staining showed that a large number of neurons exhibited atrophy, swelling and nuclear pyknosis, even died with the disappearance of Nissl bodies in the ischemic brain (Figure 4A). By contrast, IGF-1 apparently improved the neuronal survival and neuron rearrangement after HI injury, while this protective effect abrogated after the administration of PPP (Figure 4A). Furthermore, flow cytometry analysis revealed a significant increase in TUNEL^+^ neurons in the PBS-treated group post-HI compared to the control group, an effect that was markedly attenuated following IGF-1 treatment (Figure 4B). NeuN and TUNEL double staining confirmed a significant decrease in the number of TUNEL^+^ Neun^+^ cells in IGF-1-treated mice after HI (Figure 4C). Collectively, these data suggest that IGF-1 promotes neuronal survival and reduces neuronal apoptosis following HI injury.

### 3.5 IGF-1 protects neurons from apoptosis dependent on Akt signaling

**FIGURE 5:**
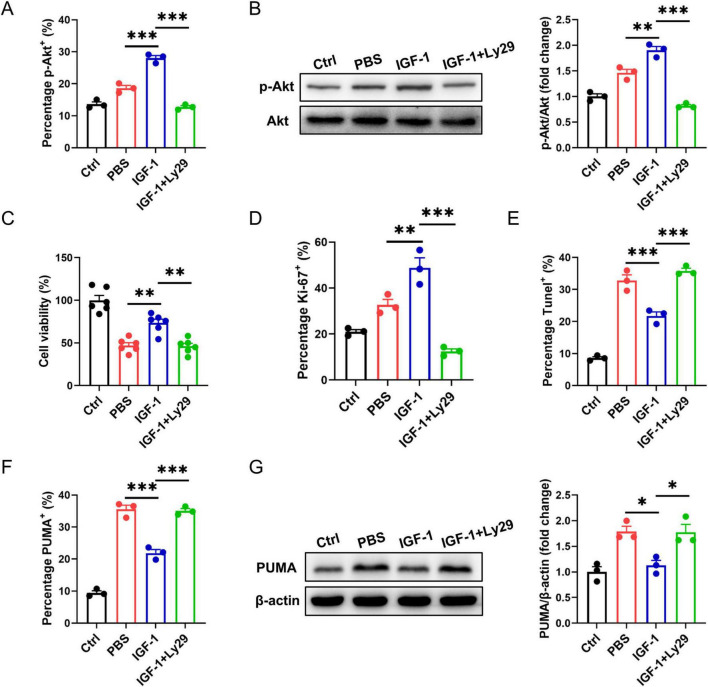
Insulin-like growth factor-1 (IGF-1) protects neurons from apoptosis dependent on AKT signaling. **(A)** Percentage of phosphorylated AKT (p-AKT) in neurons with or without PI3K inhibition at 24 h of culture with or without IGF-1 and after 3 h OGD and the culture conditions shown (*n* = 3/group). **(B)** Representative western blot images (left) and densitometric analysis (right) of p-AKT in neurons with or without PI3K inhibition at 24 h of culture with or without IGF-1 and after 3 h OGD and the culture conditions shown (*n* = 3/group). **(C)** CCK-8 assay in neurons with or without PI3K inhibition at 24 h of culture with or without IGF-1 and after 3 h OGD and the culture conditions shown (*n* = 6/group). **(D–F)** Percentage of Ki-67^+^ **(D)**, Tunel^+^ **(E)** and PUMA^+^ **(F)** neurons with or without PI3K inhibition at 24 h of culture with or without IGF-1 and after 3 h OGD and the culture conditions shown (*n* = 3/group). **(G)** Representative western blot images (left) and densitometric analysis (right) of PUMA in neurons with or without PI3K inhibition at 24 h of culture with or without IGF-1 and after 3 h OGD and the culture conditions shown (*n* = 3/group). Data are mean ± SEM. Statistical differences among multiple groups were conducted using One-way ANOVA. *P < 0.05; **P < 0.01; ***P < 0.001.

PI3K/Akt pathway is known as to be the pro-survival and proliferation cell signaling. Here, we asked whether IGF-1-mediated neuron protection involved PI3K/Akt pathway. In OGD neurons, IGF-1 treatment increased Akt phosphorylation corresponded with elevated cell survival and proliferation (Figures 5A–D). Treatment of OGD neurons with Ly294002 (Ly29), a PI3K inhibitor, blocked IGF-1-mediated Akt phosphorylation and inhibited cell survival and proliferation in response to IGF-1 (Figures 5A–D). Meantime, IGF-1 apparently reduced the percentage of TUNEL^+^ cells in OGD neurons, which was suppressed by addition of Ly29 (Figure 5E). PUMA is involved in ischemia/reperfusion-induced cerebral cell apoptosis. As shown in Figures 5F, G, the expression of PUMA was noticeably enhanced in neurons after OGD challenge. Treatment with IGF-1 inhibited OGD-induced PUMA expression in neurons, which was abrogated by addition of Ly29. Together, these data imply that IGF-1 inhibits neuron apoptosis dependent on Akt signaling.

### 3.6 Activation of Akt negatively regulates the FoxO3a/PUMA axis in OGD neurons

**FIGURE 6:**
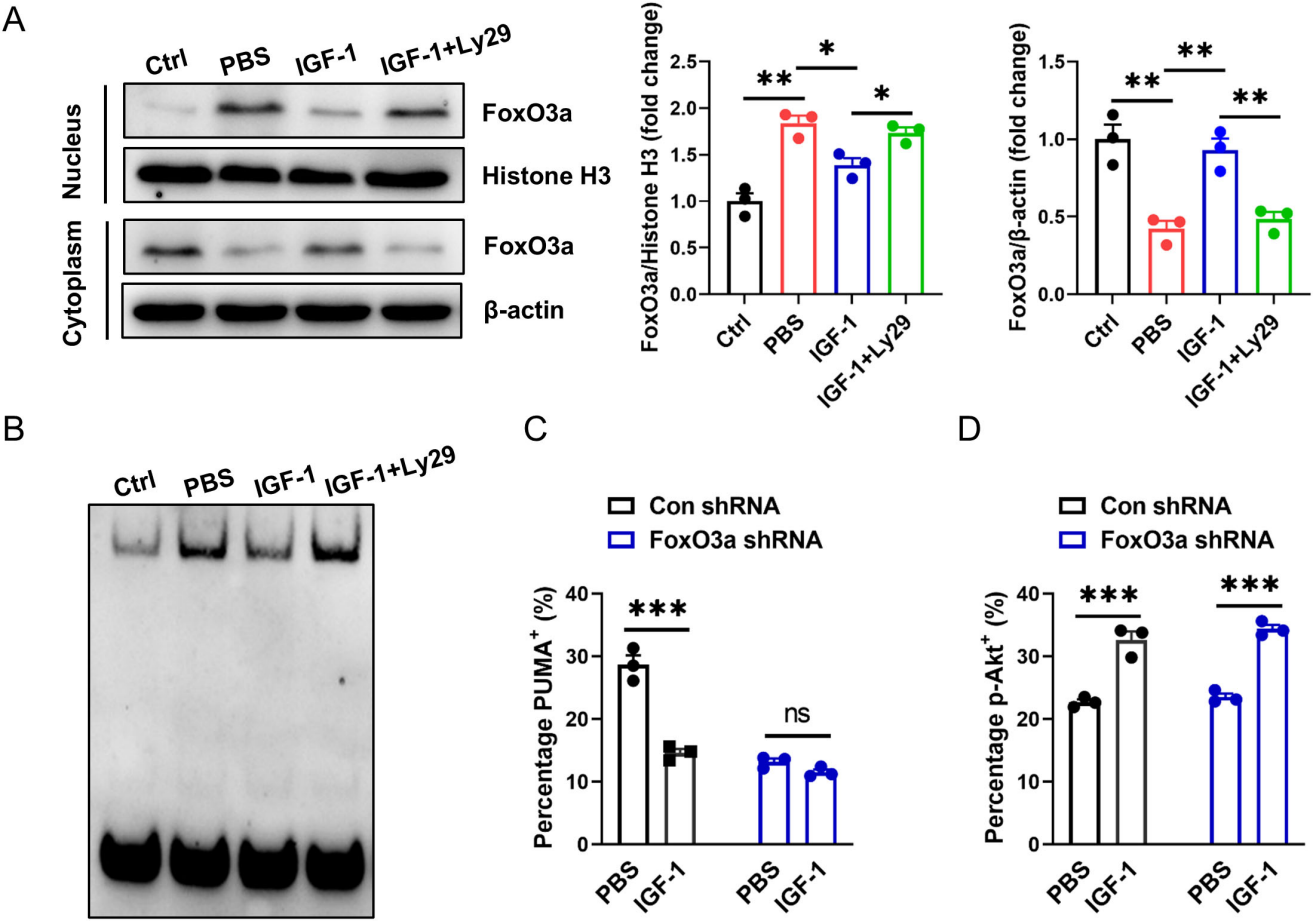
Activation of Akt negatively regulates the FoxO3a/PUMA axis in OGD neurons. **(A)** Representative western blot images (left) and densitometric analysis (right) of FoxO3a in the nucleus and cytoplasm of neurons with or without PI3K inhibition at 24 h of culture with or without IGF-1 and after 3 h OGD and the culture conditions shown (*n* = 3/group). **(B)** Electrophoretic mobility shift assay (EMSA) assay shows the direct binding of FoxO3a to PUMA gene promoter region in the nucleus of neurons with or without PI3K inhibition at 24 h of culture with or without IGF-1 and after 3 h OGD and the culture conditions shown. The graph shows results from one out of three independent experiments. **(C)** Percentage of PUMA^+^ neurons with or without FoxO3a knockdown at 24 h of culture with or without IGF-1 and after 3 h OGD and the culture conditions shown (*n* = 3/group). **(D)** Percentage of p-AKT in neurons with or without FoxO3a knockdown at 24 h of culture with or without IGF-1 and after 3 h OGD and the culture conditions shown (*n* = 3/group). Data are mean ± SEM. Statistical differences between two groups were performed using two-tailed Student’s *t*-test, while comparisons among multiple groups were performed using One-way ANOVA. *P < 0.05; **P < 0.01; ***P < 0.001; n.s., not significant.

Studies have shown that PUMA can mediate stress response as a downstream target of FoxO3a when PI3K/Akt signaling is inhibited (You et al., 2006; Zhang et al., 2015). We investigated here whether the protection of IGF-1 against neuronal death is related to inhibition of the FoxO3a/PUMA axis. As shown in Figure 6A, the amount of FoxO3a was decreased in cytoplasm, but increased in nucleus of neurons after OGD exposure. IGF-1 treatment inhibited OGD-induced FoxO3a translocation to the nucleus in neurons, which was reversed by Ly29 pretreatment (Figure 6A). Furthermore, to validate whether a protein in the nucleus binds to the PUMA gene promoter region, EMSA were performed using the nuclear extracts from every group of neurons. EMSA analysis showed the specific DNA-protein complex binding bands (Figure 6B). Moreover, the specific DNA-protein complex binding was enhanced in OGD neurons (Figure 6B). IGF-1 treatment inhibited OGD-induced increase of protein binding to PUMA promoter in the nucleus of neurons, but was reversed by Ly29-pretreated neurons (Figure 6B). Primary neurons with specific FoxO3a knockdown showed a lower percentage of PUMA^+^ cells after OGD compared to FoxO3a-expressing neurons. Notably, the percentage of PUMA^+^ neurons had no significant difference between IGF-1 and FoxO3a-siRNA treatment neurons (Figure 6C). In addition, FoxO3a knockdown did not affect Akt phosphorylation in OGD neurons, which confirmed that FoxO3a is the downstream signaling of Akt (Figure 6D). All together, these data suggest that IGF-1 activates Akt signaling, which negatively regulates the FoxO3a/PUMA axis in OGD neurons.

## 4 Discussion

Neonatal hypoxic-ischemic injury caused by perinatal asphyxia is one of the most common diseases in neonatal period (Zheng et al., 2022). There is now short of effective pharmaceutical therapeutic intervention that reduces cerebral injury or improves neurological function in infants (Yıldız et al., 2017; Liao et al., 2025a,b). In the present study, we highlight a critical astrocyte-neuron crosstalk mediated by IGF-1 in the pathological process of HIE. Our study reveals that endogenous IGF-1 is predominantly upregulated in astrocytes following HI injury, suggesting a supportive role of astrocyte-derived IGF-1 in neuronal survival. Exogenous IGF-1 supplementation further amplifies this protective astrocyte-neuron crosstalk, enhancing neuronal resilience and inhibiting apoptosis against HI stress. Notably, IGF-1-mediated antiapoptotic mechanism was dependent on suppression of the FoXO3a-PUMA signaling.

Neurons serve as the fundamental structural and functional components of the nervous system, responsible for integrating input signals and transmitting information. In the event of hypoxic-ischemic brain injury, numerous neurons are compromised, leading to neurological deficits (Hua et al., 2017). Recent evidence strongly supports the vital role of IGF-1 in promoting neuronal survival following ischemic cerebral damage, enhancing stroke outcomes and preventing additional neuronal loss (Yang et al., 2024; Lioutas et al., 2015; Zorina et al., 2023). Specifically, while ischemic stroke stimulates the proliferation and differentiation of neural progenitor cells in the subventricular zone (SVZ), the majority of these newly formed neurons perish soon after the stroke (Ceanga et al., 2021). Notably, IGF-1 has been shown to not only bolster the survival of immature neurons but also aid in their maturation within the cortical parenchyma post-stroke (Parker et al., 2017). Furthermore, recent studies have indicated the potential benefits of IGF-1 in mitigating neuronal damage, attributed to their ability to enhance mitochondrial respiratory function (Pharaoh et al., 2020; Aghanoori et al., 2021). In line with these results, our observations indicate that IGF-1 treatment safeguards neuronal survival from HI-induced brain injury by activating the pro-survival AKT signaling pathway and suppressing the pro-apoptotic pathway.

Foxo3a, a member of the FoxO family of transcription factors, is widely expressed in various human organs and tissues, and exerts regulatory effects on biological processes, such as inflammation, autophagy, apoptosis, oxidative stress, and cell cycle arrest (Fasano et al., 2019; Cao et al., 2023). One of the notable mechanisms through which FoxO3a exerts its effects is by inducing the expression of PUMA, a pro-apoptotic protein (Sun et al., 2018). PUMA, or p53 upregulated modulator of apoptosis, is a BH3-only protein that belongs to the Bcl-2 family. It functions as a critical mediator of apoptotic cell death by interacting with anti-apoptotic Bcl-2 family members, leading to the release of cytochrome c from mitochondria and subsequent activation of caspases (Shaltouki et al., 2007). Research indicates that Akt (protein kinase B) can attenuate neuronal apoptosis through phosphorylation and inhibition of caspases, pro-apoptotic Bcl-2 family molecules and selected transcription factors (FoxO3a) (Kim et al., 2014). It has been known that IGF-1 activates Akt, which inhibits Aβ-induced FoXO3a nuclear translocation, thereby reducing FoXO3a binding to the PUMA promoter and decreasing neuronal cell death (Hou et al., 2017). In agreement with these findings, our results indicated that PUMA serves as a potent executor of Foxo3a-mediated apoptosis in neurons following HI brain injury. IGF-1 administration suppresses the HI-induced upregulation of FoxO3a in neurons, and the neuroprotective effects of IGF-1 are largely attributed to the inhibition of FoxO3a-PUMA signaling.

The translational implications of our findings are twofold. First, the robust upregulation of endogenous IGF-1 in astrocytes underscores the brain’s intrinsic self-repair capacity, highlighting astrocyte-derived IGF-1 as a key component of the endogenous neuroprotective response. Second, the efficacy of exogenous IGF-1 suggests that supplementing this innate mechanism could be a viable therapeutic approach. While our findings demonstrate the therapeutic potential of IGF-1 in neonatal HIE, several limitations should be acknowledged. First, the optimal therapeutic window and dosage regimen for IGF-1 administration require further investigation. Second, the long-term effects of IGF-1 treatment on neurological recovery and potential side effects need to be evaluated in future studies. Finally, the translational applicability of our findings to clinical settings warrants validation in higher animal models.

In summary, our data indicate that IGF-1 exerts a neuroprotective effect against neonatal brain injury caused by hypoxic-ischemic conditions. It significantly reduces brain damage and behavioral impairments. Furthermore, the beneficial impact of IGF-1 is associated with enhanced neuronal survival through the downregulation of the FoxO3a-PUMA pathway. From a translational perspective, these findings not only elucidate a novel astrocyte-neuron crosstalk mechanism mediated by IGF-1 but also provide important preclinical evidence for developing IGF-1-based therapies for neonatal HIE.

## Data Availability

The original contributions presented in this study are included in this article/supplementary material, further inquiries can be directed to the corresponding authors.
